# Unlocking potential: the impact of emotional intelligence on quality of life and academic success of students with disabilities

**DOI:** 10.3389/fpsyg.2025.1659221

**Published:** 2025-09-23

**Authors:** Ibrahim A. Elshaer, Abu Elnasr E. Sobaih, Mansour Alyahya, Alaa M. S. Azazz, Mahmood A. Khan

**Affiliations:** ^1^Department of Management, School of Business, King Faisal University, Al-Ahsa, Saudi Arabia; ^2^King Salman Center for Disability Research, Riyadh, Saudi Arabia; ^3^Social Studies Department, College of Art, King Faisal University, Al-Ahsa, Saudi Arabia; ^4^Pamplin Business School, Virginia Tech, Falls Church, VA, United States

**Keywords:** emotional intelligence, university students, disabilities, quality of life, academic performance

## Abstract

**Background:**

Despite the importance of emotional intelligence (EI) for academic performance of university students with disabilities, limited research was undertaken to address this issue.

**Objectives:**

This research investigates the impact of EI on quality of life (QoL) and academic performance among university students with disabilities. Drawn on Salovey and Mayer’s EI framework, this research examines the impact of four main EI dimensions: self-emotion appraisal, others’ emotion appraisal, use of emotion, and regulation of emotion, on academic success through the lens of QoL.

**Methods:**

A quantitative, cross-sectional research design was employed, including a sample of 328 university students with several types of disabilities. Partial Least Squares Structural Equation Modeling (PLS-SEM) was employed to analyze the obtained data and test the justified hypothesized relationships.

**Results:**

The results demonstrate that the higher levels of EI are significantly related to improved QoL, which consequently has a positive impact on students’ academic performance. The results confirmed that QoL demonstrated partial mediating effects in the relationship between EI and academic achievements, signaling that EI can contribute to academic success both directly and indirectly by fostering students’ overall QoL.

**Implications:**

The study contributed to the current literature by emphasizing the interconnections of emotional competences, quality of life, and academic performance, and provided practical implications for interventions aimed at supporting this vulnerable population.

## 1 Introduction

Recently, emotional intelligence (EI) has acquired significant attention in different contexts including educational psychology, specifically regarding its potential impact on academic performance (Petersen, 2010; Bălaş-Baconschi and Dobrican, 2020; Ashori and Jalil-Abkenar, 2021; Adeskan, 2023; Adu-Amoah, 2022; Bryant, 2007; Salsabila and Adrian, 2025). EI, is conceptualized as the capacity to perceive, recognize, handle, and employee emotions in an effective way, acts a critical role in university students’ academic success (Goleman, 2005). While the traditional cognitive intelligence has usually been reviewed as the main driver of academic success, recent evidence showed that EI can contribute significantly to students’ capability to cross academic barriers, particularly among students who are facing additional challenges, i.e., students with disabilities (Sheydaei et al., 2015; Zysberg and Kasler, 2017; Suriá-Martínez et al., 2019; WenJing et al., 2021). This vulnerable population often faces several challenges in the higher education context, including physical challenges, self-stigmatization, and limited admittance to public resources, which can negatively affect quality of life (QoL) and their academic success (Al-Shaer et al., 2024). In this setting, EI may act as a pivotal determinant that can enable these students to adapt with stress, generate resilience, and sustain motivation, thus improving their academic consequences. QoL, incorporating physical-psychological health, level of autonomy, and social interaction, is another determinant element that can impact academic outcomes (Díaz and García, 2018; Dehghan et al., 2020; Adeskan, 2023; Ashori, 2025). Empirical evidence has signaled that students with disabilities repeatedly showed lower QoL levels, which can impede their academic success (Al-Shaer et al., 2024).

The mediating impact of QoL in the path from EI to academic performance is a promising area of further research, which is the focus of this research. It is suggested that EI can contribute to enhance QoL by fostering individuals’ coping strategies, social connections, and overall QoL, which consequently can positively impact academic achievements (Al-Zboon et al., 2014; Ashori and Jalil-Abkenar, 2021; Maharaj and Ramsaroop, 2022). Exploring this mediation mechanism can offer deep insights into inclusive supportive strategies for students with disabilities. This assumption was developed based on the Broaden-and-Build Theory of Positive Emotions, which was developed by Barbara Fredrickson in 1998 (Fredrickson, 2004). The theory assumes that positive emotions broaden individual’s awareness and stimulate positive actions. The theory implies that students with higher EI have a source of positive emotion and are equipped to manage negative emotion. These positive emotions would enhance QoL for students with disabilities making it as a “broadened” state, which affect acdemic performance of students with disabilities as a “built” resource. This means that QoL is a prerequisite for ensuring academic success.

Saudi Arabia (SA) has exerted intensive efforts to enhance the educational context of students with disabilities, consistent with its SA Vision’s 2030 main objectives to foster inclusivity and equity in the higher education context Saudi Vision 2030 (2025). Regardless of these efforts, there is a lack of studies that examined the intersections between EI, QoL, and academic outcomes within this vulnerable population in the SA context. Exploring these interplays is necessary for designing interventions and supportive systems that address the targeted demands of students with disabilities. Furthermore, the expected mediating role of QoL in these intersections is still underexplored. Exploring this gap is critical, as students with disabilities consistently encounter exceptional challenges that can impact both their QoL and academic achievements. The motivation for this research was derived from the recognition that students with disabilities face limitations in the higher education environment, which can influence their overall academic outcomes. Exploring the interconnections between EI, QoL, and academic performance can lead to the deigning polices and interventions for advancing inclusive higher educational surroundings that cater to the varied demands of all students. Therefore, the main aim of this paper is to investigate the critical role of EI’s several dimensions as a predictor of academic success among university students with disabilities in SA, with a specific focus on the mediating role of QoL. By exploring these interrelationships, the research aimed to provide deep insights into how EI and QoL can contribute to academic achievements, thereby enlightening policies and efforts that can contribute to the supportive practices for this vulnerable population.

To fulfill the purpose of this paper, it is started with brief introduction highlighting the research problem and its purpose. It is then followed by exploring the impact of EI on QoL and academic performance among students with disabilities. Hence, the theoretical background and hypotheses justification were explored. The research methodology adopted was then discussed including the study measures and instrument development, respondents’ selection, and employed data analysis techniques. In the results part, the paper reported statistical analyses investigating the connections between EI, QoL and academic performance. Following this, the discussion section interpreted these results and explored their theoretical and practical implications. Finally, the conclusion section summarized the whole paper and provided directions for further research avenues.

## 2 Research framework

The Salovey and Mayer's (1990) Ability Model was adopted as the theoretical framework for this study. This model conceptualized EI as the ability to “*perceive, understand, manage, and utilize emotions effectively*.” This model highlighted the main role of emotional handling in enabling cognitive efforts and containing academic duties. This model assumes that EI is a set of mental skills, which could be measured and developed. This model has four main aspects: perceiving emotions, using emotions to guide thinking, understanding emotions and finally managing emotions. This model was identified as more scientific tool to assess EI using IQ test for instance. On the other side, Goleman’s (2005) Mixed Model integrates cognitive abilities with several traits and competencies (i.e., self-awareness, empathy, social skills and motivation), which are also crucial for academic achievements.

Empirical evidence has constantly shown a positive interplay between EI and academic outcomes, signaling that EI can contribute to improving the students’ ability to handle stress, be motivated, and engaged efficiently with their studies (MacCann et al., 2020). Additionally, the Self-Determination Theory (SDT), first coined by Deci and Ryan (2013), can provide extra evidence that supports the theoretical background of the current study. The SDT is a comprehensive theory of individual motivation that highlights the critical roles of innate motivation in driving people’s behavior and actions. It suggested that people have three intrinsic psychological needs (relatedness, competence, and autonomy). These three needs, when satisfied, promote optimum functioning and QoL (Sarfo et al., 2023). In the context of higher education, SDT implies that when university students feel independent, skilled, and connected to their peers, they are more probable to be innately motivated, leading to improved academic outcomes and overall QoL (Ghaneapur et al., 2019). This is specifically relevant for students with disabilities, who may encounter extra challenges in meeting these psychological demands. The positive psychology framework can provide extra evidence in the context of our study. This framework focuses on the strengths and qualities that permit people and populations to thrive (Seligman, 2011). Within positive psychology framework, EI is considered as a driver of positive emotional practices and recovery, which can contribute to enhanced QoL and academic outcomes (Shoshani and Steinmetz, 2014).

Integrating the previous frameworks, we can hypothesis that EI can impact academic success directly and indirectly through its influence on QoL. University Students with higher level of EI are better trained to handle their emotions, leading to enhanced QoL, which consequently can improve academic performance. Additionally, fulfilling the psychological demands as explained in SDT may act as mediator in this relationship, as university students with higher level EI are more probably experienced independence, capability, and relatedness, nurturing intrinsic motivation and positive academic outcomes.

### 2.1 Emotional intelligence, quality of life, and academic performance

EI is a multidimensional construct with four interconnected yet distinctive dimensions: “Self-Emotion Appraisal (SEA), Others’ Emotion Appraisal (OEA), Use of Emotion (UOE), and Regulation of Emotion (ROE).” These four dimensions are fundamental to exploring how people can perceive, can process, and can manage emotional data, which consequently affects several aspects of personal life (Wong and Law, 2012). The first dimension of EI is SEA which describes the capability of perceiving and understanding one’s related emotions. This dimension forms the basis for efficient emotional and interpersonal connections (Salovey and Mayer, 1990; Javaid et al., 2024). This self-awareness aspect of EI permits people to mitigate stress, adapt to challenging conditions, and participate in goal-directed actions. These consequences of SEA aspect of EI are exceptionally significant for students with disabilities who frequently encounter extra academic and social challenges (Halpern, 1994; WenJing et al., 2021; Lifshitz, 2020). Goleman (2005) highlighted that SEA is crucial for personal development and QoL, as it permits people to comprehend their own emotions and adapt appropriately. Furthermore, empirical evidence has shown a positive relation between SEA and overall QoL. For example, the study conducted by Extremera and Fernández-Berrocal (2005) discovered that higher levels of perceived EI, including SEA, are correlated with higher levels of life satisfaction between higher education students. Additionally, the study by Alibabaie (2015) found a positive relationship between EI and QoL.

For students with disabilities, the competency to appraise one’s own emotions is focal in handling the extra challenges they encounter. Fields et al. (2024) emphasized that higher education students with disabilities can benefit from emotional wellness, which incorporates comprehending and handling their own emotions. This emotional ability can contribute to enhanced QoL by supporting better adapting strategies and social connections. SEA can also play a vital role in academic success (Suriá-Martínez et al., 2019). A meta-analysis study conducted by MacCann et al. (2020) argued that EI can predict positive academic outcomes, signaling that students who are skilled in understanding and handling their own emotions tend to accomplish better academic performance. Moreover, the study by Sánchez-Álvarez et al. (2016) found that EI is positively correlated with subjective QoL, which consequently can impact academic performance. Drawn on these arguments and the assumption discussed earlier from the Broaden-and-Build Theory of Positive Emotions, we can propose that:

*H1*: SEA has a significant direct impact on QoL among university students with disabilities.

*H2*: SEA has a significant direct impact on academic performance among university students with disabilities.

*H3*: SEA has a significant indirect impact on academic performance via QoL.

OEA as the second dimension of EI, describes the people’s competency to perceive and comprehend the others’ emotions. This EI aspect can facilitate effective interpersonal interaction and the structuring of supportive connection (Salovey and Mayer, 1990). This competency is exceptionally vital for students with disabilities, who may encounter different challenges in their academic life. Goleman (2005) highlighted that empathy and social competencies, intimately related to OEA, are crucial for self-satisfaction and wellness. Empirical studies have confirmed the positive correlation from OEA to QoL. For example, the study conducted by Vasiou et al. (2024) found that the appraisal of others’ emotions can significantly contribute to wellness among university students and are mediated by psychological needs satisfaction. This signaled that OEA improved QoL by meeting basic psychological needs. Likewise, research has shown that people with higher level EI, containing robust OEA competency, demonstrated better levels of QoL (Alibabaie, 2015).

OEA as a dimension of EI can play a vital role in academic success. A meta-analysis study conducted by MacCann et al. (2020) confirmed that EI can predict positive academic outcomes. While the previous study did not purposely isolate OEA, the results underscored the importance of emotional skills in academic achievement. In the context of students with disabilities, EI, principally OEA, can assist in mitigating academic challenges by promoting recovery and adaptability. These conclusions are supported by results from a study conducted by Suriá-Martínez et al. (2019), which highlighted those students with higher level of EI profiles showed better self-concept, an element that is closely related to academic success. Drawn on these arguments and the assumption discussed earlier from the Broaden-and-Build Theory of Positive Emotions, we can propose that:

*H4*: OEA has a direct significant impact on QoL among university students with disabilities.

*H5*: OEA has a direct significant impact on academic performance among university students with disabilities.

*H6*: OEA has indirect significant impact on academic performance through QoL.

UOE, as an aspect of EI, describes the capacity to employee emotions to enable people cognitive activities (i.e., critical thinking, problem-solving, and decision-taking). This competency enables people to employee their emotional states to improve motivation and performance in several duties (1990). For students with disabilities, employing emotions may be predominantly beneficial in managing academic and social problems, thereby enhancing their overall QoL and academic performance. Previous research demonstrated that higher levels of EI, containing the ability to use emotions, are corelated with high levels of QoL. For example, a study conducted by Fatima et al. (2024) highlighted a significant positive path from EI to QoL among higher education students, signaling that emotional skills can contribute effectively to high level of life satisfaction and QoL. Likewise, providing emotional support has been found to mitigate the adverse effects of metal health disorder (i.e., stress and anxiety) on QoL among students with disabilities. In the same vein, a study conducted by Al-Shaer et al. (2024) supported the previous evidence and demonstrated that emotional support can have a moderating effect in the link from mental health disorders to QoL.

The capacity to use emotions productively is also related to a high level of academic success. A meta-analysis conducted by MacCann et al. (2020) suggested that EI is a substantial predictor of academic success, emphasizing that students who can successfully control and employee their emotions tend to obtain better academic consequences. These results emphasize the key role of emotional skills in accelerating the learning and academic accomplishments. Moreover, a study conducted by Bryant (2007), Hen and Goroshit (2014), Brabcova et al. (2015), and Saber (2016) on a sample of students with learning disabilities demonstrated a positive relationship between EI and academic outcomes, signifying that emotional skills can assist students with disabilities navigate academic problems more efficiently. Drawn on these arguments and the assumption discussed earlier from the Broaden-and-Build Theory of Positive Emotions, the hypotheses below can be suggested:

*H7*: UOE has a direct significant impact on QoL among university students with disabilities.

*H8*: UOE has a direct significant impact on academic performance among university students with disabilities.

*H9*: UOE has indirect significant impact on academic performance through QoL.

ROE, as a dimension of EI, describes the skill to control and modulate emotional responses in different situations, enabling people to adapt efficiently with stress, adjust to continuously changing circumstances, and sustain emotional balance (Salovey and Mayer, 1990). This ability is exceptionally fundamental for disabled students, who frequently encounter several academic challenges. Efficient emotion regulation can contribute to the student’s ability to resilience psychologically, have strong interpersonal connections, and improve well-being, all of which are essential to QoL and academic performance (Sacks and Kern, 2008; Munday and Horton, 2021). Previous research found a positive relationship between ROE and QoL. For example, a study conducted by Gavín-Chocano et al. (2020) demonstrated that among a sample of governmental workers with physical disabilities, higher levels of emotional regulation were positively and significantly correlated with improved quality of emotions in the workplace, signaling improved QoL. Likewise, emotional regulation has been found to have a link with better mental health consequences, which are directly linked to QoL (Shoshani and Steinmetz, 2014; Lifshitz, 2020). University students who can successfully regulate their emotions are competently equipped to manage the psychological needs of academic life, causing higher levels of life satisfaction and overall well-being (Gross and John, 2003). ROE as well can play a substantial role in academic success. A meta-analysis study conducted by MacCann et al. (2020) found that EI, involving the regulation of emotions, is a considerable predictor of academic success. University students who can control their emotions are more likely to remain concentrating, manage academic stress, and endure through continuous challenges, directly leading to improved academic outcomes. Drawn on these arguments and the assumption discussed earlier from the Broaden-and-Build Theory of Positive Emotions, the following hypotheses can be proposed:

*H10*: ROE has a direct significant impact on QoL among university students with disabilities.

*H11*: ROE has a direct significant impact on academic performance among university students with disabilities.

*H12*: ROE has indirect significant impact on academic performance through QoL

### 2.2 Quality of life and academic performance

**Figure 1:**
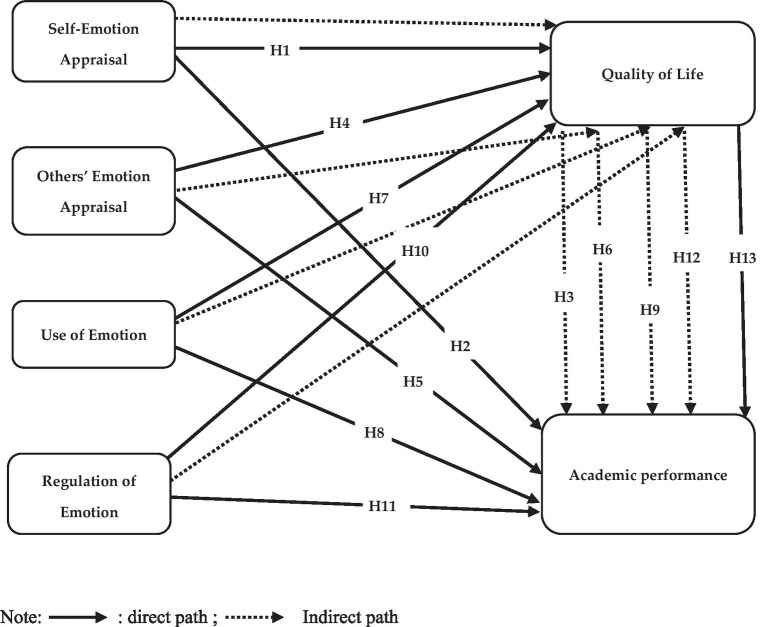
Research framework. Direct path; Indirect path.

QoL has several aspects, such as improved physical health, well psychological state, high level of independence, social connections, own beliefs, and correlation to salient attributes of the environment (World Health Organization, 1997). For university students with disabilities, these aspects can significantly impact their academic outcomes (Halpern, 1994; Torres and Vieira, 2014). A supportive and encouraging environment that improves QoL can positively influence academic outcomes. A study implemented at Qatar University evaluated the QoL of students with disabilities and correlation with academic adaptability and performance. The results indicated that higher levels of QoL were linked with better academic outcomes, which in turn can lead to improved academic performance (Al-Attiyah and Mahasneh, 2018). This signaled that improved QoL can lead to better academic performance for students with disabilities (Figure 1). Accordingly, we can propose that

*H13*: QoL has a direct significant impact on academic performance among university students with disabilities.

## 3 Methods

### 3.1 Study measures and the instrument development

The measures and variables employed were based on an extensive review of previous literature to select reliable and valid scales. To operationalize emotional intelligence (EI), the Wong and Law Emotional Intelligence Scale (WLEIS) (Wong and Law, 2012) was employed. WLEIS is a commonly considered self-report scale developed to measure emotional intelligence (EI) with four distinct and related dimensions (self-emotion appraisal, others’ emotion appraisal, use of emotion, and regulation of emotion). The scale has 16 items, four items for each EI dimension. Participants are asked to rate each item on a scale with a 7-point scale ranging from 1 “Strongly Disagree” to 7 “Strongly Agree.” Although the current study was conceptually directed from Salovey and Mayer’s (1990) ability model of emotional intelligence (EI), the Wong and Law Emotional Intelligence Scale (WLEIS) was used in the operationalization process. While performance-based scale (i, e. “the Mayer–Salovey–Caruso Emotional Intelligence Test” -MSCEIT) are more closely consistent with the ability model, they are regularly encountering a high cost, long time-intensiveness, and less practicable in large-scale or cross-section field research (Joseph and Newman, 2010; O’Connor et al., 2019). Conversely, the WLEIS was explicitly advanced employing the four-dimensions of the ability model “self-emotion appraisal, others’ emotion appraisal, use of emotion, and regulation of emotion” and has been widely validated in Middle Eastern and Asian settings (Law et al., 2004). Furthermore, meta-analytic studies confirmed the reliability and predictive validity of self-report EI measures, including WLEIS, in educational and organizational context (Miao et al., 2017). Moreover, the WLEIS has been widely revealed to be manageable, concise, and easy to administer, which is mainly beneficial when investigating populations that may require accessibility conditions (i.e., students with disabilities). Previous research papers have successfully used WLEIS among vulnerable or distinct samples, including people with health challenges and students that require inclusive educational context, arguing that the scale still reliable and valid in these cases (Chan, 2004; Extremera and Rey, 2016). Therefore, WLEIS was considered both theoretically adequate and culturally suitable for the current study.

Likewise, academic performance was operationalized by 3 items as suggested by Owusu-Acheaw and Larson (2015) and was found to be valid and reliable in several previous empirical studies (Elshaer et al., 2025; Zayed et al., 2024; Sobaih et al., 2022). Finally, QoL was operationalized by five items that reflected the “Satisfaction with Life Scale” (SWLS). This SWLS was developed by Diener et al. (1985) and described the perception of people’s cognitive judgments regarding overall life satisfaction. To ensure the face validity of the developed scale, the questionnaire was first reviewed by 10 academics who assessed the clarity, suitability and relevance, of the written questions. Likewise, a pilot test was implemented with 10 disabled King Faisal University (KFU) students. The replies from these two stages indicated that the questions were clearly readable and understood, with the minimum language modifications. These two steps ensured that the questionnaire showed adequate content and face validity. The full questionnaire was structured into 3 key parts. The initial section provided the targeted respondents with a clear and brief introduction that explained the study’s objectives, and to obtain a consent form to approve voluntary participation and guarantee the ethical criteria. The second section was structured to obtain main demographic information, such as type of participants’ disability, age, enrolled academic year, and respondents’ gender. The final part includes the study’s main latent factors. The study scale was originally established in English. To safeguard their appropriateness for usage in SA context, a translation and back-translation technique was conducted. First, 2- bilingual specialists separately translated the scale items into Arabic. Following, 2 other bilingual specialists, blinded to the first original versions, and back translated the scale items into English. Inconsistencies between the first original and the second back-translated forms were discussed, and very minor wording changes were made consequently.

### 3.2 Population and sampling

The General Population and Housing Census conducted in 2024 in KSA stated that around 2% (1.8%) of the total population, representing around 64,800 residents, suffered from some type of disability. The different types of disabilities are hearing disability, mobility limitations, cognitive abilities, visual impairments, self-care challenges, and communication challenges. Conspicuously, higher education students have a significant ratio of this demographic, representing around 58% that are fully enrolled KSA public universities: King Abdulaziz University (1,569 full time students), King Saud University (663), Taibah University (523), Umm Al-Qura University (381), and King Faisal University (330). A convenience sampling approach was selected to collect the required data, and a statistical power analysis with G*Power 3.1 program was conducted to detect the minimum sample size. The results yielded that a minimum of 124 respondents was required to represent the total population adequately. The calculation was based on an effect size (f^2^ = 0.15), with 5 predictors, and a significance p level of 0.05, and a requested power of 0.95. To facilitate data collection, 30 well trained enumerators were recruited to adequately adopt the ethical research policies, involving obtaining the informed consent form and ensuring the participant’s confidentiality. Out of 550 questionnaires distributed, 328 were retained to be valid, with a response rate of 59%. Invalid and incomplete forms were not analyzed and archived. The final dataset has roughly balanced gender distribution (52% female, 48% male) and students age was ranged from 17 to 24 years old; the highest percentage of students was suffered from mobility disability (50%) following visual impairment (23%), hearing impairments (15%) other disabilities (12%) accordingly. As the same participant answered the dependent and independents questions, common method variance (CMV) might be an issue (Lindell and Whitney, 2001). Therefore, we adopted several procedural and statistical remedies to make sure that CMV is not an issue in this research. The procedural steps included adopting various questions formats and order and respecting participants’ anonymity. The statistical remedies adopted with SPSS, Exploratory Factor Analysis (EFA) option, we exposed all items to “Harman’s Single Factor Test” to find out the amount of the variance that can be extracted in one single dimension, the result yielded a value of 41% variance extracted, indicating that CMV is not a problem in our dataset (Podsakoff et al., 2003).

This research followed the ethical guidelines of the Declaration of Helsinki. The research was approved by the Deanship of Scientific Research Ethical Committee, King Faisal University (project number: KFU-2025-ETHICS2820, date of approval: 6 September 2024). These guidelines were followed during the research design, data collection and data interpretation to protect the privacy of the respondents.

## 4 Research methods

This study utilized a cross-sectional survey approach employing a structured questionnaire to test the assumed relationships among the study dimensions. This approach is considered adequate as it enabled the gathering of data from a large sample size within a fairly short period of time and allowed the investigation of relationships within PLS-SEM data analysis techniques (Hair et al., 2021).

### 4.1 Data analysis technique and study results

In our study, data analysis was guided by employing “Partial Least Squares Structural Equation Modeling” (PLS-SEM) with SmartPLS 4 software. The choice of this technique (PLS-SEM) was considered for several methodological advantages (Hair et al., 2021). It has several advantages over covariance-based SEM. It has fewer and less stringent data requirements compared to covariance-based SEM. First, given that our study is exploratory in its nature and has complex relationships between observed and latent variables, PLS-SEM is particularly adequate for assessing and predicting interrelationships between the study constructs (Leguina, 2015). Second, PLS-SEM has no rigorous assumptions about data distribution, making it adequate for non-normally distributed datasets (Hair et al., 2021). This merit makes PLS-SEM beneficial when dealing with real-world data that regularly demonstrates non-normal distributions (Chin, 1998). Third, PLS-SEM provides robust abilities for assessing and calculating mediating impact in the structural models (Leguina, 2015). This advantage is critical for understanding the mechanisms through which independent dimensions impact factors variables through mediators. Finally, compared to the commonly and widely used data analysis techniques that are based on covariance (i.e., CB-SEM), PLS-SEM is less sensitive to issues such as model identification problems, hence improving the reliability of the analytical results (Hair et al., 2017).

Table 1 presented the correlation matrix to inspect the relationships among all scale items. All values were in the expected directions, and none were excessively high or low (> 0.9 or <0.3), indicating that multicollinearity is improbably to be an issue in our study. Furthermore, multicollinearity was tested using Variance Inflation Factor (VIF). As shown in Table 2, all VIF scores ranged between 2.7 and 1.5, confirming the absence of multicollinearity concern (Tabachnick and Fidell, 2019) and consequently did not bias the path coefficients or inflate the standard errors. Additionally, Table 2 showed the descriptive statistics (means and standard deviations) of the study variables as well and demonstrated a proper variability and were within the predictable theoretical range of the scale items. Furthermore, to test the distribution of the data (normality), skewness and kurtosis scores were inspected. All scores were found to be within the adequate range of −2 to +2, signifying that the data did not deviate significantly from normality (Tabachnick and Fidell, 2019). Following the suggestions provided by Leguina (2015), the analysis process was conducted in two succeeding stages. The measurements model psychometric properties (Table 2) were inspected in stage one, while in stage two, the structural model was evaluated regarding hypothesis assessment.

**Table 1:** Pearson’s correlation coefficients.

SEA_1	SEA_2	SEA_3	SEA_4	OEA_1	OEA_2	OEA_3	OEA_4	UOE_1	UOE_2	UOE_3	UOE_4	QOL1	QOL2	QOL3	QOL4	QOL5	Perf_1	Perf_2	Perf_3	ROE_1	ROE_2	ROE_3	ROE_4
1	0.7^**^	0.6*^**^	0.5^**^	0.4^**^	0.4^**^	0.4^**^	0.4^***^	0.4^**^	0.4^**^	0.5^**^	0.5^**^	0.5^**^	0.4^**^	0.4^**^	0.4^**^	0.4^**^	0.4^**^	0.5^**^	0.5^**^	0.4^**^	0.4^**^	0.5^**^	0.5^**^
	1	0.6^***^	0.5^**^	0.4^**^	0.4^**^	0.4^**^	0.4^**^	0.5^**^	0.4^**^	0.5^**^	0.5^**^	0.5^**^	0.4^**^	0.4^**^	0.4^**^	0.4^**^	0.4^**^	0.5^**^	0.5^**^	0.5^**^	0.5^**^	0.5^**^	0.5^**^
		1	0.5^**^	0.3^**^	0.3^**^	0.3^**^	0.3^**^	0.5^**^	0.4^**^	0.5^**^	0.5^**^	0.4^**^	0.4^**^	0.4^**^	0.5^**^	0.4^**^	0.5^**^	0.4^**^	0.4^**^	0.5^**^	0.4^**^	0.5^**^	0.4^**^
			1	0.4^**^	0.4^**^	0.4^**^	0.3^**^	0.4^**^	0.3^**^	0.4^**^	0.5^**^	0.5^**^	0.4^**^	0.3^**^	0.4^**^	0.4^**^	0.3^**^	0.3^**^	0.3^**^	0.3^**^	0.2^**^	0.4^**^	0.4^**^
				1	0.5^**^	0.5^**^	0.4^**^	0.4^**^	0.3^**^	0.4^**^	0.4^**^	0.4^**^	0.4^**^	0.3^**^	0.4^**^	0.4^**^	0.4^**^	0.4^**^	0.3^**^	0.4^**^	0.3^**^	0.4^**^	0.5^**^
					1	0.7^**^	0.6^***^	0.3^**^	0.3^**^	0.4^**^	0.4^**^	0.4^**^	0.5^**^	0.4^**^	0.4^**^	0.4^**^	0.4^**^	0.4^**^	0.4^**^	0.4^**^	0.3^**^	0.3^**^	0.5^**^
						1	0.6^***^	0.3^**^	0.3^**^	0.4^**^	0.4^**^	0.4^**^	0.5^***^	0.4^**^	0.4^**^	0.4^**^	0.4^**^	0.4^**^	0.4^**^	0.4^**^	0.4^**^	0.4^**^	0.5^**^
							1	0.3^**^	0.3^**^	0.3^**^	0.4^**^	0.4^**^	0.5^**^	0.3^**^	0.3^**^	0.3^**^	0.4^**^	0.4^**^	0.4^**^	0.3^**^	0.3^**^	0.3^**^	0.5^**^
								1	0.6*^**^	0.5^**^	0.5^**^	0.4^**^	0.5^**^	0.4^**^	0.4^**^	0.2^**^	0.4^**^	0.4^**^	0.4^**^	0.4^**^	0.4^**^	0.5^**^	0.5^**^
									1	0.5^**^	0.5^**^	0.4^**^	0.3^**^	0.4^**^	0.4^**^	0.2^**^	0.4^**^	0.4^**^	0.3^**^	0.4^**^	0.3^**^	0.4^**^	0.4^**^
										1	0.5^**^	0.4^**^	0.5^**^	0.5^**^	0.5^***^	0.3^**^	0.4^**^	0.5^**^	0.3^**^	0.3^**^	0.4^**^	0.5^**^	0.4^**^
											1	0.6^***^	0.6^***^	0.4^**^	0.4^**^	0.4^**^	0.5^**^	0.5^**^	0.4^**^	0.4^**^	0.3^**^	0.5^**^	0.5^**^
												1	0.6^***^	0.4^**^	0.3^**^	0.4^**^	0.4^**^	0.4^**^	0.3^**^	0.3^**^	0.3^**^	0.4^**^	0.5^**^
													1	0.4^**^	0.4^**^	0.3^**^	0.5^**^	0.5^**^	0.5^**^	0.4^**^	0.4^**^	0.4^**^	0.5^**^
														1	0.5^**^	0.4^**^	0.3^**^	0.4^**^	0.3^**^	0.3^**^	0.4^**^	0.5^**^	0.4^**^
															1	0.5^**^	0.5^**^	0.4^**^	0.4^**^	0.3^**^	0.5^**^	0.5^**^	0.5^**^
																1	0.5^**^	0.4^**^	0.4^**^	0.4^**^	0.4^**^	0.5^**^	0.4^**^
																	1	0.5^**^	0.5^**^	0.5^**^	0.4^**^	0.5^**^	0.5^**^
																		1	0.5^**^	0.4^**^	0.5^**^	0.5^**^	0.5^**^
																			1	0.6^**^	0.5^**^	0.5^**^	0.5^**^
																				1	0.5^**^	0.5^**^	0.5^**^
																					1	0.6^***^	0.5^**^
																						1	0.6^***^
																							1

***: Correlations significant at 0.001;** Correlations significant at 0.01; SEA_1-SEA_4: items employed to measure Self-Emotion Appraisal; OEA_1-OEA_4: items employed to measure Others’ Emotion Appraisal; UOE_1-UOE_4: items employed to measure Use of Emotion; QoL_1-QoL_5: items employed to measure quality of life; Perf_1-Perf_3: item employed to meaure Academic Performance; ROE_1-ROE_4: items employed to measure Regulation of Emotion.

**Table 2:** Factor loadings and other psychometric properties.

Factors/ Items	F. L.	α	C. R.	A. V. E	V. I. F.	M	S. D.	Skewness	Kurtosis
Self-Emotion Appraisal		0.881	0.889	0.741					
SEA_1	0.926				2.7	2.8	1.2	−0.234-	−1.384-
SEA_2	0.932				2.3	2.8	1.2	−0.203-	−1.382-
SEA_3	0.805				1.7	3.0	1.2	−0.187-	−1.141-
SEA_4	0.767				1.6	3.1	1.0	−0.297-	−0.571-
Others’ Emotion Appraisal		0.887	0.892	0.752					
OEA_1	0.770				1.5	3.4	1.2	−0.496-	−0.665-
OEA_2	0.940				1.6	3.4	1.2	−0.507-	−0.745-
OEA_3	0.938				1.5	3.4	1.2	−0.507-	−0.742-
OEA_4	0.807				1.8	3.5	1.1	−0.832-	0.009
Use of Emotion		0.840	0.845	0.675					
UOE_1	0.827				1.8	3.2	1.3	−0.485-	−0.871-
UOE_2	0.807				1.8	3.3	1.2	−0.491-	−0.883-
UOE_3	0.829				1.8	3.3	1.3	−0.336-	−1.088-
UOE_4	0.824				1.7	3.3	1.3	−0.371-	−0.954-
Regulation of Emotion		0.853	0.855	0.694					
ROE_1	0.817				1.8	3.1	1.2	−0.081-	−0.931-
ROE_2	0.818				1.8	3.3	1.1	−0.217-	−0.850-
ROE_3	0.864				2.2	3.2	1.2	−0.240-	−0.939-
ROE_4	0.832				1.9	3.3	1.2	−0.433-	−0.941-
Quality of Life		0.819	0.822	0.580					
QOL1	0.767				1.8	3.3	1.1	−0.728-	−0.070-
QOL2	0.775				1.7	3.4	1.2	−0.625-	−0.501-
QOL3	0.760				1.7	3.5	1.2	−0.610-	−0.527-
QOL4	0.756				1.7	3.6	1.2	−0.742-	−0.226-
QOL5	0.749				1.6	3.5	1.0	−0.505-	−0.061-
Academic Performance		0.792	0.792	0.706					
Acd_Perf_1	0.849				1.7	3.5	1.2	−0.557-	−0.623-
Acd_Perf_2	0.845				1.7	3.4	1.1	−0.323-	−0.935-
Acd_Perf_3	0.826				1.5	3.3	1.1	−0.406-	−0.682-

F. L., Factor Loading; α, Cronbach Alpha; C. R., Composite reliability; A. V. E., Average Variance Extracted; V. I. F., Variance Inflation Factor; M, Mean; S. D., Standard Deviation.

### 4.2 Stage number 1: measurement model inspection

The evaluation of the study measurement model encompassed assessment of convergent validity, discriminant validity, and reliability. All the study factor loadings are above 0.70, consistent with the threshold recommended by Fornell and Larcker (1981) and demonstrating high items’ reliability. Furthermore, both Cronbach’s alpha and composite reliability (C. R.) scores exceeded the value of 0.70, indicating adequate internal consistency (Gerbing and Anderson, 1988). The average variance extracted (AVE) results for each factor surpassed the value of 0.50, signaling a satisfactory convergent validity (see Table 2). Discriminant validity was evaluated employing the Fornell-Larcker metric, which requires that the square root of each factor’s AVE to be higher than its intercorrelations with other factors. All factors fulfilled this criterion, signaling adequate discriminant validity (see Table 3). Moreover, the heterotrait-monotrait ratio (HTMT) was inspected as per Henseler et al. (2009) suggestions, with all HTMT scores remaining below the 0.85 threshold to confirm discriminant validity (see Table 4). The cross-loadings values shown in Table 5 further confirmed the scale discriminant validity, where each item is highly loaded to its predetermined factor with no cross-loadings. These outcomes confirm the reliability and validity of the employed measurement model.

**Table 3:** “Fornell and Larcker.”

Factors/ Items	1	2	3	4	5	6
1-AP	**0.840**					
2-OEA	0.605	**0.867**				
3-QoL	0.723	0.660	**0.761**			
4-ROE	0.768	0.580	0.721	**0.833**		
5-SEA	0.648	0.549	0.701	0.667	**0.861**	
6-UOE	0.638	0.578	0.755	0.668	0.707	**0.822**

AP, Academic Performance; OEA, Others’ Emotion Appraisal; QoL, Use of Emotion; ROE, Regulation of Emotion; SEA, Self-Emotion Appraisal; UOE, Use of Emotion.Bold values indicates the square roots of AVEs.

**Table 4:** “Heterotrait-monotrait ratio” (HTMT) - Matrix.

Factors/ Items	1	2	3	4	5	6
1-AP						
2-OEA	0.724					
3-QoL	0.789	0.772				
4-ROE	0.735	0.666	0.757			
5-SEA	0.773	0.624	0.726	0.764		
6-UOE	0.777	0.665	0.797	0.783	0.716	

AP, Academic Performance; OEA, Others’ Emotion Appraisal; QoL, Use of Emotion; ROE, Regulation of Emotion; SEA, Self-Emotion Appraisal; UOE, Use of Emotion.

**Table 5:** Factor cross loadings.

Factors/ Items	AP	OEA	QoL	ROE	SEA	UOE
Acd_Perf_1	**0.849**	0.537	0.639	0.641	0.523	0.549
Acd_Perf_2	**0.845**	0.519	0.632	0.618	0.565	0.585
Acd_Perf_3	**0.826**	0.469	0.552	0.677	0.545	0.475
OEA_1	0.524	**0.770**	0.544	0.508	0.519	0.532
OEA_2	0.536	**0.940**	0.605	0.515	0.466	0.501
OEA_3	0.542	**0.938**	0.609	0.534	0.481	0.512
OEA_4	0.494	**0.807**	0.526	0.450	0.436	0.456
QOL1	0.467	0.541	**0.767**	0.471	0.586	0.628
QOL2	0.649	0.576	**0.775**	0.562	0.529	0.654
QOL3	0.451	0.458	**0.760**	0.535	0.469	0.562
QOL4	0.597	0.462	**0.756**	0.599	0.557	0.582
QOL5	0.562	0.466	**0.749**	0.571	0.524	0.434
ROE_1	0.654	0.451	0.508	**0.817**	0.534	0.502
ROE_2	0.615	0.424	0.582	**0.818**	0.515	0.500
ROE_3	0.640	0.452	0.653	**0.864**	0.604	0.619
ROE_4	0.650	0.599	0.650	**0.832**	0.567	0.594
SEA_1	0.595	0.496	0.628	0.615	**0.926**	0.622
SEA_2	0.604	0.515	0.621	0.632	**0.932**	0.648
SEA_3	0.578	0.425	0.601	0.596	**0.805**	0.628
SEA_4	0.439	0.452	0.563	0.436	**0.767**	0.527
UOE_1	0.535	0.458	0.597	0.579	0.562	**0.827**
UOE_2	0.453	0.405	0.527	0.499	0.519	**0.807**
UOE_3	0.529	0.481	0.645	0.562	0.597	**0.829**
UOE_4	0.567	0.539	0.692	0.549	0.632	**0.824**

AP, Academic Performance; OEA, Others’ Emotion Appraisal; QoL, Use of Emotion; ROE, Regulation of Emotion; SEA, Self-Emotion Appraisal; UOE, Use of Emotion.Bold values indicates item loading to its factor.

### 4.3 Stage number 2: structural model evaluation

**Figure 2:**
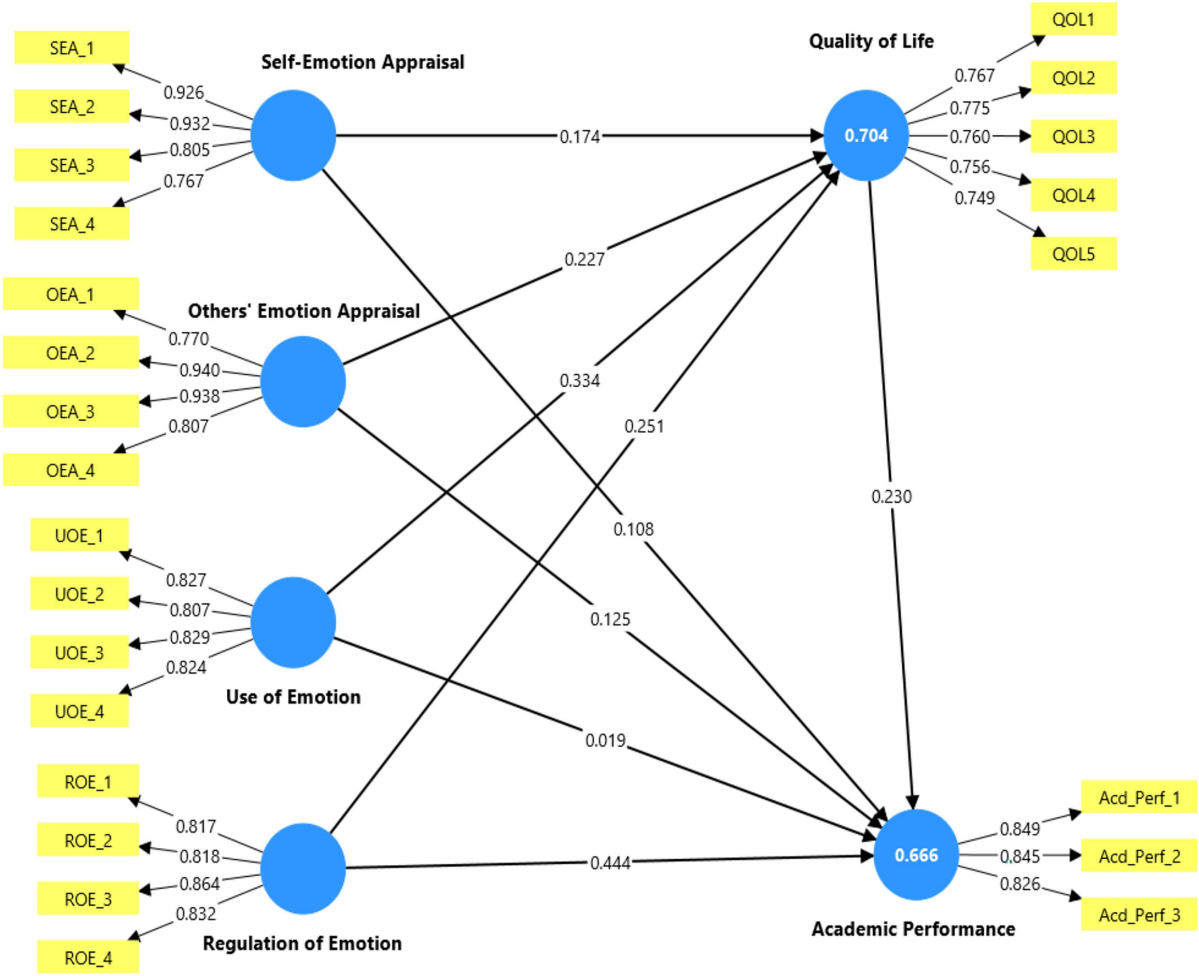
The research model.

Several key criteria can be employed to evaluate the structural model predictive and explanatory capacities, as suggested by Chin (2010). The Stone-Geisser’s Q^2^ value, reported in the blindfolding analysis, was inspected to test the model’s predictive relevance. All endogenous factors showed Q^2^ scores above zero, specifically, QoL (0.693), and academic performance satisfaction (0.637), signaling that the study model can predict these factors effectively. Explanatory power was assessed via each endogenous factor’s coefficient of determination (R^2^) values. The R^2^ scores were strong for QoL (0.704), and academic performance (0.666), suggesting that the study model can explain a large proportion of the variance in QoL and academic performance. The Model goodness of fit was further evaluated employing the “Standardized Root Mean Square Residual” (SRMR), the SRMR value was 0.067, which is below the suggested maximum value of 0.08, signaling a minimum discrepancy among the observed and predicted data. The NFI value (0.926) exceeded the recommended cut off point 0.90 and signaling a good GoF of the tested model and inferring that the specified structural hypothesized model adequately captures the underlying tested relationships among the study factors. These results confirm that the study’s structural model has robust predictive power, good explanatory power, and a satisfactory overall model fit, thus validating its capability for hypothesis testing (Figure 2).

After testing the reliability and validity of the developed measurement model and the designed structural model, the hypothesis testing phase can be initiated. As shown in Table 6 the path coefficient values, and their related t-values were extracted employing a bootstrapping approach with 5, 000 samples. Additionally, the significance of indirect paths was evaluated using the bootstrapping procedure with 5,000 resamples and a 95% bias-corrected confidence interval. This non-parametric method was chosen because it did not assume normality of indirect paths.

**Table 6:** Hypotheses testing and related *t* and *p* values.

Path coeffecients	*β*	*T*	*p*	Outcomes
Self-Emotion Appraisal - > Quality of Life	0.174	3.253	0.001	H1: Supported
Self-Emotion Appraisal - > Academic Performance	0.108	1.972	0.049	H2: Supported
Slelf-Emotion Appraisal - > Quality of Life - > Academic Performance	0.040	2.793	0.005	H3: Supported
Others’ Emotion Appraisal - > Quality of Life	0.227	6.220	0.000	H4: Supported
Others’ Emotion Appraisal - > Academic Performance	0.125	2.599	0.009	H5: Supported
Others’ Emotion Appraisal - > Quality of Life - > Academic Performance	0.052	3.490	0.000	H6: Supported
Use of Emotion - > Quality of Life	0.334	7.363	0.000	H7: Supported
Use of Emotion - > Academic Performance	0.019	0.333	0.739	H8: Rejected
Use of Emotion - > Quality of Life - > Academic Performance	0.077	3.432	0.001	H9: Supported
Regulation of Emotion - > Quality of Life	0.251	6.022	0.000	H10: Supported
Regulation of Emotion - > Academic Performance	0.444	7.252	0.000	H11: Supported
Regulation of Emotion - > Quality of Life - > Academic Performance	0.058	3.519	0.000	H12: Supported
Quality of Life - > Academic Performance	0.230	4.367	0.000	H13: Supported

The PLS-SEM results indicated that self-emotional appraisal (as a dimension of emotional intelligence) was found to have a direct positive influence on student QOL (*β* = 0.174, *t* = 3.253, *p* < 0.01) and academic performance (*β* = 0.108, *t* = 1.972, *p* < 0.05), and a positive indirect impact on academic performance through QoL (*β* = 0.040, *t* = 2.793, *p* < 0.01) supporting H1, H2 and H3. Additionally, others’ emotion appraisal (as a dimension of emotional intelligence) showed a positive and significant direct impact on students’ QoL (*β* = 0.227, *t* = 6.220, *p* < 0.001), and academic performance (*β* = 0.125, *t* = 2.599, *p* < 0.01), and a positive indirect impact on academic performance through QoL (*β* = 0.052, *t* = 3.490, *p* < 0.001) this confirming H4, H5, and H6. Similarly, use of emotions (as a dimension of emotional intelligence) successfully impacted QOL directly (*β* = 0.334, *t* = 7.363, *p* < 0.001), supporting H7, but failed to significantly influence student academic performance (*β* = 0.019, *t* = 0.333, *p* = 0.739), thus H8 was rejected, but was found to indirectly influence academic performance through QOL (*β* = 0.077, *t* = 3.432, *p* < 0.001), supporting H9. As presented in Table 6, hypotheses number 10. 11, and 12 were also supported as regulation of emotions (as a dimension of EI) was found to have direct impact on disabled student QoL (*β* = 0.251, *t* = 6.002, *p* < 0.001) and academic performance (*β* = 0.444, *t* = 7.252, *p* < 0.001) and indirect impact on academic performance through QoL (*β* = 0.058, *t* = 3.519, *p* < 0.001). Finally, the direct impact of student QOL on academic performance showed a positive and significant path coefficient (*β* = 0.230, *t* = 4.367, *p* < 0.001), which supports H13. All the direct and indirect paths as shown in Table 6 are significant (except the path from use of emotion to academic performance) inferring a partial mediation effect.

## 5 Discussion

The results of this research highlighted the focal role of EI in improving not only QoL but also the academic performance of the students with disabilities in KSA. The positive significant associations that were detected between several EI dimensions and the students’ QoL and academic achievements highlighted the multifaceted advantages of EI in educational context. Notably, SEA positively impacted both QoL and academic performance. This proposes that university students who are skilled at identifying and understanding their self-emotions are capable of equipping to mitigate the challenges related to their disabilities, resulting in enhanced overall life satisfaction and academic achievements. These results are consistent with previous evidence that reported self-awareness as a critical dimension of EI that can contribute to superior psychological adjustment and academic success (Saber, 2016). The results are also aligned with Goleman’s (2005) study, which asserted that self-awareness is crucial to emotional intelligence, enabling people to better recognize and understand their own emotions, hence facilitating the adoption of coping strategies in the decision-creation processes. In the context of KSA universities, where disabled students might face societal and infrastructural challenges, the capability to appraise own emotions can result in improved psychological recovery and academic success. Likewise, the second dimension of EI, others’ OEA was found to have a positive relationship with QoL and academic performance. This aspect of EI mirrors the capability to understand and perceive others’ emotions, which is crucial for helpful interpersonal communications and social inclusion. In the context of KSA universities, where an inclusive learning environment is being progressively considered, the capability to connect with colleagues, peers and faculty members can significantly improve the learning experience of students with disabilities (Ragmoun and Alfalih, 2024).

Interestingly, UOE, the third employed dimension of EI, strongly and significantly impacted QoL but failed to significantly influence the students’ academic performance. This result indicated that while the capacity to exploit emotions can improve student’ well-being, it cannot directly be translated to improved academic achievements. This discrepancy can be explained by some external factors such as the ease of use of the university academic support services and accommodations, which can play a key role in the academic performance of students with disabilities (WenJing et al., 2021). Additionally, the fourth employed aspect of EI, ROE significantly influenced QoL and academic performance. Successful emotion regulation can enable university students to handle stress and remain focused, which are critical for good academic outcomes and overall QoL. These results are consistent with previous studies emphasizing the importance of ROE in adapting with the exceptional challenges encountered by university students with disabilities in the educational settings (Ashori and Jalil-Abkenar, 2021). Likewise, the observed direct significant and positive influence of QoL on academic performance highlights the interdependence of QoL and educational success. University students with a higher QoL are more likely to participate efficiently in academic life, implying that interventions aimed at improving QoL can favor academic success.

The PLS-SEM report also showed some evidence regarding the specific indirect effects. The results showed that QoL can significantly mediate the impacts of all four dimensions EI on academic performance. While UOE did not demonstrate a significant direct impact on academic performance, it positively impacted QoL, which consequently impacted academic success. This result highlighted the key intermediary role of QoL between EI and academic performance. This mediation effects confirm the assumption made by the Broaden-and-Build Theory of Positive Emotions (Fredrickson, 2004),that the QoL for students with disabilities is a “broadened” state, which not only have a direct effect on academic performance of such students but also it could make the effect of positive emotion coming from EI more strength on academic performance of these students with disabilities. This highlights the value of QoL is a prerequisite for ensuring academic success of these students, which has to gain more attention from higher education administrators to ensure the academic success of their students.

University students with disabilities frequently face unique circumstances that can influence their emotional welfare and academic performance. The students’ abilities to appraise and regulate their emotions (SEA and ROE) can improve the overall QoL by improving recovery strategies, which drive academic success and engagement. These results are aligned with previous evidence that EI can contribute to a higher psychological well-being and academic achievement level through improved student self-efficacy and resilience (Shengyao et al., 2024). The specific results that UOE can indirectly influence academic performance via QoL suggested that the capability to control emotions positively influences students’ overall QoL, improving their academic outcomes. This indirect path emphasized the significant role of emotional application in the academic environment, specifically for students who suffer from disabilities who regularly employ emotional resources to mitigate academic barriers. Additionally, the significant mediating effects of QoL in the path from OEA to academic performance indicated that the aptitude to understand others’ emotions can improve student social interactions, leading to better QoL and, in turn, higher academic performance. This result is consistent with the previous literature that highlighted the significant role of the social dimensions of EI and their influence on educational consequences (Brabcova et al., 2015; Elshaer, 2023).

## 6 Conclusions, limitations, and future research

This study explored the impacts of the four aspects of Emotional Intelligence EI (SEA, OEA, UOE, and ROE) on academic performance, with QoL acting as a mediator among university students with disabilities in KSA. The findings highlighted the key role of EI in improving this vulnerable student population’s QoL and academic achievements. The results indicated that all four dimensions of EI can positively impact QoL, consequently improving academic outcomes. Conspicuously, while UOE failed to directly and significantly foster academic performance, its positive impact on QoL enabled the indirect effects on academic success. This highlights the significant role of QoL as an instrument through which emotional capabilities are transferred into academic achievements.

Aligning with the goals of KSA Vision 2030, which highlights inclusive learning and the empowerment of people with disabilities, specifically it aligned with the “Human Capability Development Program,” which highlights the inclusive education and the empowerment of people with disabilities. By signifying the significant key role of EI in improving academic success and QoL, the study highpoints a practical pathway for attaining Vision 2030 objectives. Additionally, the current study results have several significant implications. Including EI development procedures in the higher education context could act as a strategic program to strengthen the QoL and academic success of students with disabilities. Such programs will be consistent with the national educational agenda and promote a deep, inclusive, supportive academic context. Additionally, decision makers in KSA universities should apply comprehensive support approaches centered on academic performance and fostering students’ life satisfaction and social integration. This holistic system can lead to a high level of academic achievements, principally for students with disabilities who may face extra barriers. Policymakers should also consider models that combine EI training and QoL programs into the educational environment to facilitate equal opportunities for academic success among all segments of students.

As highlighted earlier this research adopted self-reporting survey using convenience sampling from students with disabilities, hence, there are some limitations of the research due to its cross-sectional and sampling design. Further research avenues should explore the long-term impacts of EI on QoL and academic success of students with disabilities. A longitudinal research approach can offer a better understanding of the sustainability of such relationships and their impact over time. Additionally, expanding the model to include various educational contexts, socioeconomic factors, environmental, pedagogical factors and student segments can improve the generalizability of results and inform best procedures in inclusive education. Furthermore, this paper did not investigate the diversity within the student population in terms of disability type, severity, or the level of support received. These variables may moderate the tested relationships. Future research should include them as moderating variables, which would offer a wider insight into the precise requirements and strengths of diverse groups of students with disabilities. Finally, although the current study revealed positive associations between EI, QoL, and AP among university students with disabilities, it is important to acknowledge that the efficiency of EI interventions in improving academic consequences in this specific population needs more empirical evidence. Consequently, while our results suggest a potential pathway for intervention, future empirical evidence is needed to assess the influence of targeted EI programs within the SA context.

## Data Availability

The raw data supporting the conclusions of this article will be made available by the authors, without undue reservation.

## References

[ref1] Adeskan A. (2023). Emotional intelligence and academic performance of students with learning disability. Int. J. Acad. Multidiscip. Res. 6, 149–154.

[ref2] Adu-Amoah P. (2022). Academic self-efficacy, emotional intelligence, personality traits on academic performance of teacher-trainees with special needs in Ghana. Ghana: University of Cape Coast.

[ref3] Al-Attiyah A. Mahasneh R. (2018). Quality of life of Qatar University students with disability and its relation to their academic adjustment and performance. ERIC 33, 562–557.

[ref4] Alibabaie N. (2015). A study on the relationship between quality of life, emotional intelligence and life satisfaction among students. Health Educ. Health Prom. 3, 3–13.

[ref5] Al-Shaer E. A. Aliedan M. M. Zayed M. A. Elrayah M. Moustafa M. A. (2024). Mental health and quality of life among university students with disabilities: the moderating role of religiosity and social connectedness. Sustainability 16:644. doi: 10.3390/su16020644

[ref6] Al-Zboon E. Ahmad J. F. Theeb R. S. (2014). Quality of Life of Students with Disabilities Attending Jordanian Universities. Int. J. Spec. Educ. 29, 1–8.

[ref7] Ashori M. (2025). Effect of emotional intelligence on the general health and life orientation of adolescents who have a hearing impairment. Int. J. Disabil. Dev. Educ. 72, 282–294. doi: 10.1080/1034912X.2024.2355342

[ref8] Ashori M. Jalil-Abkenar S. S. (2021). Emotional intelligence: quality of life and cognitive emotion regulation of deaf and hard-of-hearing adolescents. Deafness Educ. Int. 23, 84–102. doi: 10.1080/14643154.2020.1766754

[ref9] Bălaş-Baconschi C. Dobrican L.-D. (2020). Emotional intelligence and its influence on the adaptive skills of children with hearing disabilities. Educatia 21, 85–92. doi: 10.24193/ed21.2020.19.10

[ref10] Brabcova D. Zarubova J. Kohout J. Jošt J. Kršek P. (2015). Effect of learning disabilities on academic self-concept in children with epilepsy and on their quality of life. Res. Dev. Disabil. 45, 120–128. doi: 10.1016/j.ridd.2015.07.018 26233763

[ref11] Bryant H. C. (2007). The relationship between emotional intelligence and Reading comprehension in high school students with learning disabilities. Berrien Springs, MI: Andrews University.

[ref12] Chan D. W. (2004). Perceived emotional intelligence and self-efficacy among Chinese secondary school teachers in Hong Kong. Pers. Individ. Dif. 36, 1781–1795. doi: 10.1016/j.paid.2003.07.007

[ref13] Chin W. W. (1998). The partial least squares approach to structural equation modeling. Adv. Hosp. Leis. 295, 295–336.

[ref14] Chin W. W. (2010). “How to write up and report PLS analyses” in Handbook of partial least squares. eds. Esposito Vinzi V. Chin W. W. Henseler J. Wang H. (Berlin, Heidelberg: Springer Berlin Heidelberg), 655–690.

[ref15] Deci E. L. Ryan R. M. (2013). Intrinsic motivation and self-determination in human behavior. Cham: Springer Science & Business Media.

[ref16] Dehghan F. Kaboudi M. Alizadeh Z. Heidarisharaf P. (2020). The relationship between emotional intelligence and mental health with social anxiety in blind and deaf children. Cogent Psychol. 7:1716465. doi: 10.1080/23311908.2020.1716465

[ref17] Díaz M. G. García M. J. (2018). Emotional intelligence, resilience and self-esteem in disabled and non-disabled people. Enfermería Glob. 17, 263–273. doi: 10.6018/eglobal.17.2.291381

[ref18] Diener E. Emmons R. A. Larsen R. J. Griffin S. (1985). The satisfaction with life scale. J. Pers. Assess. 49, 71–75. doi: 10.1207/s15327752jpa4901_13, PMID: 16367493

[ref19] Elshaer I. A. (2023). Front-line hotel employees mental health and quality of life post COVID-19 pandemic: the role of coping strategies. Heliyon 9:e16915. doi: 10.1016/j.heliyon.2023.e16915, PMID: 37287607 PMC10234689

[ref20] Elshaer I. A. AlNajdi S. M. Salem M. A. (2025). Sustainable AI solutions for empowering visually impaired students: the role of assistive technologies in academic success. Sustainability 17:5609. doi: 10.3390/su17125609

[ref21] Extremera N. Fernández-Berrocal P. (2005). Perceived emotional intelligence and life satisfaction: predictive and incremental validity using the trait meta-mood scale. Pers. Individ. Differ. 39, 937–948. doi: 10.1016/j.paid.2005.03.012

[ref22] Extremera N. Rey L. (2016). Attenuating the negative impact of unemployment: the interactive effects of perceived emotional intelligence and well-being on suicide risk. PLoS One 11:e0163656. doi: 10.1371/journal.pone.0163656, PMID: 27685996 PMC5042532

[ref23] Fatima S. Khan M. L. Kousar R. (2024). Emotional intelligence, religiosity and quality of life among university students. J. Soc. Organiz. Matt. 3, 455–471. doi: 10.56976/jsom.v3i2.94

[ref24] Fields A. M. Lewis O. Castle M. Smith-Hill R. B. Stinnett C. V. (2024). College students with intellectual and developmental disabilities’ experiences, conception, and development of emotional wellness. Intellect. Dev. Disabil. 62, 274–286. doi: 10.1352/1934-9556-62.4.274, PMID: 39069300

[ref25] Fornell C. Larcker D. F. (1981). Evaluating structural equation models with unobservable variables and measurement error. J. Mark. Res. 18, 39–50. doi: 10.1177/002224378101800104

[ref26] Fredrickson B. L. (2004). The broaden-and-build theory of positive emotions. Phil. Trans. R. Soc. B or Philos. Trans. R. Soc. B 359, 1367–1377. doi: 10.1098/rstb.2004.1512, PMID: 15347528 PMC1693418

[ref27] Gavín-Chocano O. Molero D. García-Martínez I. (2020). Relationship between life satisfaction, burnout and emotional intelligence among professionals who work directly with people with intellectual disabilities. Electron. J. Res. Educ. Psychol. 18, 425–446. doi: 10.25115/ejrep.v18i52.3080

[ref28] Gerbing D. W. Anderson J. C. (1988). An updated paradigm for scale development incorporating unidimensionality and its assessment. J. Mark. Res. 25, 186–192. doi: 10.1177/002224378802500207

[ref29] Ghaneapur M. Eftekhar H. Montazeri A. Garmarudi G. Yaseri M. Ahvanoei A. R. (2019). Effectiveness of a self-determination theory (SDT) based intervention on physical activity, quality of life, and happiness: a protocol for a randomized clinical trial. J. Biochem. Technol. 10, 108–117.

[ref30] Goleman D. (2005). Emotional intelligence: Why it can matter more than IQ. New York: Bantam.

[ref31] Gross J. J. John O. P. (2003). Individual differences in two emotion regulation processes: implications for affect, relationships, and well-being. J. Pers. Soc. Psychol. 85, 348–362. doi: 10.1037/0022-3514.85.2.348, PMID: 12916575

[ref32] Hair J. Hair J. F. Hult G. T. M. Ringle C. M. Sarstedt M. (2021). A primer on partial least squares structural equation modeling (PLS-SEM). New York: Sage publications.

[ref33] Hair J. Hollingsworth C. L. Randolph A. B. Chong A. Y. L. (2017). An updated and expanded assessment of PLS-SEM in information systems research. Ind. Manag. Data Syst. 117, 442–458. doi: 10.1108/IMDS-04-2016-0130

[ref34] Halpern A. S. (1994). Quality of life for students with disabilities in transition from school to adulthood. Soc. Indic. Res. 33, 193–236. doi: 10.1007/BF01078962

[ref35] Hen M. Goroshit M. (2014). Academic procrastination, emotional intelligence, academic self-efficacy, and GPA: a comparison between students with and without learning disabilities. J. Learn. Disabil. 47, 116–124. doi: 10.1177/0022219412439325, PMID: 22442254

[ref36] Henseler J. Ringle C. M. Sinkovics R. R. (2009). The use of partial least squares path modeling in international marketing. Adv. Inter. Market. 20, 277–319. doi: 10.1108/S1474-7979(2009)0000020014

[ref37] Javaid Z. K. Mubashar M. Mahmood K. Noor A. Javed N. Akhtar K.. (2024). Effect of emotional intelligence and self-concept on academic performance: a systematic review of cross-cultural research. Bull. Bus. Econ. 13, 189–199. doi: 10.61506/01.00315

[ref38] Joseph D. L. Newman D. A. (2010). Emotional intelligence: an integrative meta-analysis and cascading model. J. Appl. Psychol. 95, 54–78. doi: 10.1037/a0017286, PMID: 20085406

[ref39] Law K. S. Wong C. S. Song L. J. (2004). The construct and criterion validity of emotional intelligence and its potential utility for management studies. J. Appl. Psychol. 89, 483–496. doi: 10.1037/0021-9010.89.3.483, PMID: 15161407

[ref40] Leguina A. (2015). A primer on partial least squares structural equation modeling (PLS-SEM). Int. J. Res. Method Educ. 38, 220–221. doi: 10.1080/1743727X.2015.1005806

[ref41] Lifshitz H. (2020). “Affect and emotional intelligence in populations with intellectual disability” in Growth and development in adulthood among persons with intellectual disability. ed. Lifshitz H. (Cham: Springer International Publishing), 253–301.

[ref42] Lindell M. K. Whitney D. J. (2001). Accounting for common method variance in cross-sectional research designs. J. Appl. Psychol. 86, 114–121. doi: 10.1037/0021-9010.86.1.114, PMID: 11302223

[ref43] MacCann C. Jiang Y. Brown L. E. Double K. S. Bucich M. Minbashian A. (2020). Emotional intelligence predicts academic performance: a meta-analysis. Psychol. Bull. 146, 150–186. doi: 10.1037/bul0000219, PMID: 31829667

[ref44] Maharaj P. Ramsaroop A. (2022). Emotional intelligence as a contributor to enhancing educators’ quality of life in the COVID-19 era. Front. Psychol. 13:921343. doi: 10.3389/fpsyg.2022.921343, PMID: 36072055 PMC9443812

[ref45] Miao C. Humphrey R. H. Qian S. (2017). A meta-analysis of emotional intelligence and work attitudes. J. Occup. Organ. Psychol. 90, 177–202. doi: 10.1111/joop.12167

[ref46] Munday P. Horton C. (2021). Emerging scholar: the impact of emotional intelligence among children with disabilities and the role of professional educators and caregivers: a literature review. Int. J. Whole Child 6, 91–107.

[ref47] O’Connor P. J. Hill A. Kaya M. Martin B. (2019). The measurement of emotional intelligence: a critical review of the literature and recommendations for researchers and practitioners. Front. Psychol. 10:1116. doi: 10.3389/fpsyg.2019.01116 31191383 PMC6546921

[ref48] Owusu-Acheaw M. Larson A. G. (2015). Use of social media and its impact on academic performance of tertiary institution students: a study of students of Koforidua polytechnic, Ghana. J. Educ. Pract. 6, 94–101.

[ref49] Petersen V. C. (2010). The relationship between emotional intelligence and middle school students with learning disabilities. Teaneck, NJ: Fairleigh Dickinson University.

[ref50] Podsakoff P. M. MacKenzie S. B. Lee J.-Y. Podsakoff N. P. (2003). Common method biases in behavioral research: a critical review of the literature and recommended remedies. J. Appl. Psychol. 88, 879–903. doi: 10.1037/0021-9010.88.5.879, PMID: 14516251

[ref51] Ragmoun W. Alfalih A. A. (2024). Inclusive special needs education and happiness of students with physical disabilities in Saudi Arabia: the role of school satisfaction and self-concept. Educ. Sci. 14:209. doi: 10.3390/educsci14020209

[ref52] Saber M. (2016). Emotional intelligence, Psychological adjustment and academic achievement among female students with and without social learning difficulties in Najran. Int. J. Learn. Dev. 6, 121–143.

[ref53] Sacks G. Kern L. A. (2008). Comparison of quality of life variables for students with emotional and behavioral disorders and students without disabilities. J. Behav. Educ. 17, 111–127. doi: 10.1007/s10864-007-9052-z

[ref54] Salovey P. Mayer J. D. (1990). Emotional intelligence. Imagin. Cogn. Pers. 9, 185–211. doi: 10.2190/DUGG-P24E-52WK-6CDG

[ref55] Salsabila N. D. Adrian Y. (2025). Emotional intelligence and family support in parents' acceptance of children with special needs. Nusantara J. Behav. Soc. Sci. 4, 35–42. doi: 10.47679/njbss.202576

[ref56] Sánchez-Álvarez N. Extremera N. Fernández-Berrocal P. (2016). The relation between emotional intelligence and subjective well-being: a meta-analytic investigation. J. Posit. Psychol. 11, 276–285. doi: 10.1080/17439760.2015.1058968

[ref57] Sarfo J. O. Obeng P. Kyereh H. K. Ansah E. W. Attafuah P. Y. A. (2023). Self-determination theory and quality of life of adults with diabetes: a scoping review. J. Diabetes Res. 2023, 1–12. doi: 10.1155/2023/5341656, PMID: 37091043 PMC10115521

[ref58] Saudi Vision 2030. (2025) Saudi Vision 2030. Available online at: https://www.vision2030.gov.sa/en (Accessed 24 May 2025).

[ref59] Seligman M. E. (2011). Flourish: a visionary new understanding of happiness and well-being. New York: Simon and Schuster.

[ref60] Shengyao Y. Salarzadeh Jenatabadi H. Mengshi Y. Minqin C. Xuefen L. Mustafa Z. (2024). Academic resilience, self-efficacy, and motivation: the role of parenting style. Sci. Rep. 14:5571. doi: 10.1038/s41598-024-55530-7, PMID: 38448465 PMC10918079

[ref61] Sheydaei M. Adibsereshki N. Movallali G. (2015). The effectiveness of emotional intelligence training on communication skills in students with intellectual disabilities. Int. J. Develop. Disabil. 62:2047387715Y.000. doi: 10.1179/2047387715Y.0000000014

[ref62] Shoshani A. Steinmetz S. (2014). Positive psychology at school: a school-based intervention to promote adolescents’ mental health and well-being. J. Happiness Stud. 15, 1289–1311. doi: 10.1007/s10902-013-9476-1

[ref63] Sobaih A. E. E. Hasanein A. Elshaer I. A. (2022). Higher education in and after COVID-19: the impact of using social network applications for e-learning on students’ academic performance. Sustainability 14:5195. doi: 10.3390/su14095195

[ref64] Suriá-Martínez R. Ortigosa Quiles J. M. Riquelme Marin A. (2019). Emotional intelligence profiles of university students with motor disabilities: differential analysis of self-concept dimensions. Int. J. Environ. Res. Public Health 16:4073. doi: 10.3390/ijerph16214073, PMID: 31652742 PMC6862470

[ref65] Tabachnick B. G. Fidell L. S. (2019). Using multivariate statistics. Boston, MA, USA: Pearson.

[ref66] Torres V. M. F. Vieira S. C. M. (2014). Quality of life in adolescents with disabilities. Rev. CEFAC 16, 21–37. doi: 10.20971/kcpmd.2024.67.3.21

[ref67] Vasiou A. Vasilaki E. Mastrothanasis K. Galanaki E. (2024). Emotional intelligence and university students’ happiness: the mediating role of basic psychological needs’ satisfaction. Psychol. Int. 6, 855–867. doi: 10.3390/psycholint6040055

[ref68] WenJing L. TongYao Y. Kim D.-J. (2021). A study on the influence of emotional intelligence on life satisfaction of people with disabilities in China. Health Welfare 23, 163–181. doi: 10.23948/kshw.2021.12.23.4.163

[ref69] Wong C.-S. Law K. S. (2012). Wong and Law emotional intelligence scale. London: Sage.

[ref70] World Health Organization (1997). WHOQOL: measuring quality of life. Geneva: World Health Organization.

[ref71] Zayed M. A. Moustafa M. A. Elrayah M. Elshaer I. A. (2024). Optimizing quality of life of vulnerable students: the impact of physical fitness, self-esteem, and academic performance: a case study of Saudi Arabia universities. Sustainability 16:4646. doi: 10.3390/su16114646

[ref72] Zysberg L. Kasler J. (2017). Learning disabilities and emotional intelligence. J. Psychol. 151, 464–476. doi: 10.1080/00223980.2017.1314929, PMID: 28494197

